# Study protocol: Worldwide comparison of vitamin D status of immigrants from different ethnic origins and native-born populations—a systematic review and meta-analysis

**DOI:** 10.1186/s13643-019-1123-4

**Published:** 2019-08-22

**Authors:** Said Yousef, Jesse Elliott, Douglas Manuel, Ian Colman, Manny Papadimitropoulos, Alomgir Hossain, Nathalie Leclair, George A. Wells

**Affiliations:** 1 School of Epidemiology and Public Health, Faculty of Medicine, University of Ottawa, Ottawa, ON Canada; 2 Cardiovascular Research Methods Centre, University of Ottawa Heart Institute, Ottawa, ON Canada; 3 Ottawa Hospital Research Institute, Ottawa, ON Canada; 4 Institute for Clinical Evaluative Sciences, Ottawa and Toronto, ON Canada; 5 Department of Family Medicine, University of Ottawa, Ottawa, ON Canada; 6 Canadian Institutes of Health Research, Ottawa, ON Canada; 7 Eli Lilly Canada Inc, Toronto, ON Canada; 8 Faculty of Pharmacy, University of Toronto, Toronto, ON Canada; 9 Berkman Library, University of Ottawa Heart Institute, Ottawa, ON Canada

**Keywords:** Systematic review, Meta-analysis, Vitamin D, Immigrants’ health deterioration

## Abstract

**Background:**

A growing body of literature indicates that, worldwide, immigrants experience health deterioration after their arrival into their adopted country, and moreover, they have lower vitamin D compared to the native-born population. We plan to review if the levels of vitamin D are comparable between different ethnic groups in different regions of the world with those of native-born populations and to identify the possible associations between vitamin D deficiency and disease status among immigrants.

**Methods/design:**

A systematic review and meta-analysis will be conducted following the methods of the Cochrane handbook for systematic reviews. A literature search was performed to identify studies on immigrants and vitamin D. The primary outcome is vitamin D levels, and the secondary outcome is any vitamin D deficiency-related disease. Study design and participant characteristics will be extracted, including ethnicity, country of birth and/or origin, and the host country. Descriptive and meta-analytic summaries of the outcomes will be derived. Distiller-SR and RevMan will be used respectively for data management and meta-analysis.

**Discussion:**

This systematic review may partially help clarify vitamin D-related health deterioration in migrants; moreover, to develop a global guideline that specifies sub-populations, in which the evidence and vitamin D-related recommendations might differ from the overall immigrant population.

**Systematic review registration:**

PROSPERO CRD42018086729

**Electronic supplementary material:**

The online version of this article (10.1186/s13643-019-1123-4) contains supplementary material, which is available to authorized users.

## Background

Healthy people are more likely to migrate, and those with serious medical conditions may be disqualified [1, 2]. Despite particular groups, such as refugees, who are not as healthy as other immigrants, substantial evidence indicates that the health of immigrants at the time of arrival is significantly better than the health of the native-born population of the host country [3–8]. Recent studies have shown that the physical and mental health of immigrants declines after their arrival into the adopted country [2, 4, 8–12], and health changes can occur within 5 to 10 years [9, 13–15]. Migration studies suggest that the integration process is likely a new experience for immigrants, in which changes in the environment and lifestyle are well-documented factors predisposing migrants to chronic diseases [8, 16, 17]. Moreover, migration experience and post-migration integrations are believed to explain the decline in the health status of immigrants [4, 11, 18]. However, the mechanistic reasons underlying the decline in immigrants’ health have not been explicitly identified [4, 14]. Worldwide research evidence suggests that migration is a significant risk factor for low vitamin D levels due to resettlement changes in lifestyle and sun exposure [19–22]. Vitamin D plays a crucial role in physiological functions; it regulates the absorption of calcium and phosphorus that support both skeletal (bone hemostasis) and non-skeletal health [23, 24].

The dietary sources for vitamin D are plant-based foods (e.g., mushroom) which include vitamin D2 (ergocalciferol) and the animal-based foods (e.g., salmon and tuna fish) which include vitamin D3 (cholecalciferol) [21, 25]. In addition to the natural food-based sources, the human body obtains vitamin D from artificial sources such as supplements and fortified food. The primary source, however, is through skin exposure to the sunlight (cutaneous synthesis of vitamin D) [26], and therefore, vitamin D is commonly known as the ‘sunshine vitamin’ because the body can make it through exposure to sunlight whereas most other vitamins are food-based ingestion [27]. However, the ability of body to create and maintain sufficient levels affected by socio-demographic factors (e.g., socioeconomic status, age, and gender), geographical and environmental factors (e.g., season and the latitude), cultural and religious factors (e.g., clothing, sedentary behaviors and prolonged breastfeeding time), lifestyle and dietary factors (e.g., vegetarian diet), as well as genetic and health factors (e.g., skin pigmentation and obesity) [20, 23, 28–30]. A study on people from different ethnic origin who have similar practices proposes that skin pigmentation is possibly the most significant risk factor for vitamin D deficiency irrespective of the ultraviolet light exposure [31].

The concentration of 25-hydroxyvitamin D serum (25(OH)D) represents the combined contributions of the cutaneous synthesis and the dietary intake of vitamin D and is considered the best clinical indicator for the overall adequacy of vitamin D [23, 25, 27]. There is no international agreement regarding the reference range for serum 25(OH) D concentrations. The Institute of Medicine (IOM) has developed different categories for the concentration levels of the serum 25(OH)D in blood, which include optimal (> 75 nmol/l), sufficient (> 50 nmol/l), inadequate or deficient (≤ 50 nmol\l), and deficient (< 30 nmol/l) levels [27]. According to the World Health Organization, the levels of serum 25(OH)D below 50 nmol/l were classified as insufficient and levels below 25 nmol/l were considered as deficient [32].

Vitamin D deficiency is a global pandemic health problem in all age groups that occurs in countries with both high and low levels of sunlight (degree of the latitudes) [23, 24]. Hilger et al. in a systematic review of studies from 44 countries with data from 112 articles (168,389 participants), reported a wide range of serum 25(OH)D between 4.9 and 136.2 nmol/l with age and sex variations. Mean levels of < 50 nmol/l were reported in one third of these studies [33]. The highest levels of serum 25(OH)D were among participants who live in North America while those who live in the Middle East and Africa regions have much lower levels [33].

Studies show that worldwide migrant populations, including refugees and asylum, have lower levels of vitamin D compared with the local populations [20, 30, 34]. Refugees are considered at particularly higher risk for vitamin D deficiency due to staying indoors to avoid dangers, cold weather, living in high-rise buildings that limit their outdoor activities and sun exposure, and concerns regarding skin cancer [20]. The age, ethnicity, country of origin, higher latitudes, nutritional barriers, cultural practices, and the length of time after immigration were reported in several studies as being associated with the risk of vitamin D deficiency among immigrants [20, 30, 36]. Migrants are often considered as low-SES individuals, and immigrants of low SES were found to have a higher risk of vitamin D deficiency [35], as well as to develop physical and mental illnesses [16, 30, 36].

Previous studies support the hypothesis that the inadequate or deficient level of vitamin D is associated with increased risk of all-cause mortality and a wide range of physical and mental health diseases, including osteoporosis, type 1 diabetes mellitus, cancer, cardiovascular disease, obesity, schizophrenia and depression, and the metabolic syndrome [17, 23, 33, 36–39].

Although there is disagreement about optimal levels of serum 25(OH)D, evidence suggests that levels < 30 nmol/l are associated with an increased risk of some diseases in which vitamin D deficiency has been found to play a role. Moreover, the thresholds for serum 25(OH)D may vary according to different outcomes and subgroups, and the most advantageous levels begin at 75 nmol/l [40–43].

The most relevant systematic review to this protocol published by Martin et al. 2016 has focused on dark skin immigrants including both first- and second-generation immigrants. Moreover, the included studies were selected from certain regions associated with dark skin, namely, Africa; West, South, Central, and Southeast Asia; the Middle East; the Caribbean, and Central America [20]. Whereas the current systematic review will include only the first-generation immigrants who were defined as foreign-born population and no geographical restrictions will be applied. However, to the best of our knowledge, no study has systematically reviewed the vitamin D deficiency-related diseases and immigrant status.

The aims of this systematic review and meta-analysis are the following: first is to compare the levels of vitamin D between ethnic groups of immigrants in different regions with those of native-born populations. Consequently, the findings will be used to establish global maps for vitamin D status of immigrants and non-immigrants, as well as maps for the same ethnic immigrants (at high risk) in different geographical regions of the world. Second is to identify the possible associations between vitamin D deficiency and disease status among immigrants.

## Methods/design

The protocol of this review has been registered on PROSPERO (CRD42018086729) [44]. The strategy involves a comprehensive systematic review of the literature and meta-analysis. The procedures will follow the methods outlined in the Cochrane handbook for systematic reviews [45]. This includes guidance in planning the review, searching and selecting studies, collecting data, as well as the risk of bias and prospective meta-analysis. The protocol of this systematic review follows the Preferred Reporting Items for Systematic Reviews and Meta-Analyses Protocol (PRISMA-P) Statement [46]; the checklist is presented in Additional file 1.

### Literature search

A systematic literature search was performed to identify studies on immigrants and vitamin D. The primary search strategy was completed in MEDLINE(R) ALL (Ovid) by a medical information specialist using a combination of subject headings and keywords. The strategy was peer-reviewed by an independent information specialist using the Peer Review of Electronic Search Strategies (PRESS) guidelines [47]. The final search strategy was run on August 16, 2018, and translated to EMBASE Classic + Embase (Ovid) (available in Additional file 2), PubMed, and Web of Science Indexes (SCI-EXPANDED, SSCI, A&HCI, CPCI-S, CPCI-SSH, ESCI) on the same day, and to Cochrane (Wiley on INSERT DATE). With regard to the grey literature, Dissertations and Theses Global (ProQuest) was searched as well as international and governmental websites, including the World Health Organization (WHO), the Canadian Institute for Health Information (CIHI), and the National Institute for Health Care Excellence (NICE). Supplemental searches will be performed using backward chaining (looking through the bibliographies of included studies for additional relevant articles).

### Eligibility criteria and study selection

Two reviewers independently will follow the population, exposure, comparator, and outcomes (PECO) criteria in evaluating the included studies. The population of interest is immigrants, defined as foreign-born. The exposure is the integration process over time in the new environment after immigration, and the comparator is the non-immigrant (native-born population). The primary outcome is vitamin D levels, and the secondary outcome is any vitamin D deficiency-related disease (e.g., cardiovascular, diabetes, cancer, osteoporosis, depression, chronic pain, and respiratory infections).

All study designs will be eligible except the following publication types: case reports, case series, systematic or narrative reviews, editorials, and commentaries. Studies will be excluded if they do not define or include migrant status, including populations at higher risk for vitamin D deficiency such as chronic illness, and if the concentration levels of vitamin D are not reported. The publication with the largest sample size will be included if the same population is presented in more than one publication. No studies will be excluded from the search based on the language and the study date.

All studies will be independently screened and evaluated for eligibility by two reviewers, and any disagreement will be discussed and resolved by consensus.

### Data extraction and synthesis

The data will be extracted by two independent reviewers. If necessary, discrepancies will be resolved through discussion [48]. Standardized and piloted forms for data extraction will be used to extract the characteristics of the studies and participants, as well as the specific details from each study (e.g., ethnicity, country of birth and/or origin, and the host country). Data for all immigrants and a priori defined subgroups will be extracted. Based on the availability, the mean and/or the median of the concentration levels of vitamin D will be summarized in a tabular format, pooled via meta-analysis, and used to compare immigrants to non-immigrants. Descriptive statistics will be implemented for the baseline features of the included studies. The vitamin D status will be identified based on the IOM categories (optimal, adequate, insufficient, or deficient) [27]. When the concentration level of vitamin D is given as nanograms per milliliter, it will be multiplied by 2.496 to attain the nanomoles per liter [49]. Participant and study characteristics of the included studies will be used to assess the clinical and methodological heterogeneity, respectively. The heterogeneity between the included studies will be completed through a visual assessment of forest plots and the calculation of the *I*^2^ statistic, which provides a numerical summary of heterogeneity. The following categories of *I*^2^ statistic will be considered: < 25%, 25 to 50%, and 50 to 75%. For *I*^2^ > 50, heterogeneity will be explored using subgroup and meta-regression. If the reason for heterogeneity is identified, it will be reported accordingly. If heterogeneity is not resolved, the results will be pooled for *I*^2^ between 50 and 75%, and a caveat regarding the heterogeneity will be provided. For *I*^2^ > 75%, the results will not be pooled. Outcome data will be pooled using a random-effects model. Distiller SR and RevMan will be used to complete data extraction and meta-analyses, respectively.

### Subgroup and sensitivity analysis

Depending on the availability and the quality of data (risk of bias), subgroup analysis will be conducted based on age, sex, ethnicity, country of birth and/or origin, geographical location and the latitude of the host country for immigrants, season, the immigration class (family, economic, and refugee classes), and the time since immigration.

### Risk of bias and quality evaluation

The quality of the included studies will be assessed using the relevant tools depending on the study design. These tools may include Joanna Briggs, Scottish Intercollegiate Guidelines Network-Publication no.50 (SIGN50), Risk Of Bias In Non-randomized Studies of Exposure (ROBINS-E), as well as Cochrane Collaboration’s Risk of Bias tool (ROB v. 2.0) [45, 50–52]. Two independent reviewers will evaluate the quality, and any disagreements between reviewers will be discussed and resolved by consensus.

## Discussion

The baseline risk for vitamin D deficiency is higher among migrants especially those who have darker skin, such as Middle Eastern and African populations, and those who migrate from equatorial region to northern latitude [20, 33, 53]. Cultural and lifestyle practices that minimize sun exposure also increase the risk in these subgroups [20]. However, the developed recommendations for vitamin D supplementation provided in the Dietary Reference Intakes (DRIs) still inadequately address the growing epidemic of vitamin D insufficiency in immigrants and refugees [49]. For instance, the DRIs for vitamin D and calcium sets the recommended of daily intake assuming minimal sun exposure for all populations. Moreover, no additional recommendations are given for sub-populations such as immigrants living in high northern latitudes, those with darker skin pigmentation, or those who wear heavy clothing that inhibits sun exposure [54, 55]. In addition, most of the worldwide guidelines on vitamin D and/or immigrants’ health does not entirely address these subpopulations in their recommendations [56–61].

Nonetheless, recent reviews recommended future studies to assess relevant data on vitamin D and determinants, including lifestyle factors with a subgroup comparison of the population within the same country [33], as well as further research specific to migrant populations to establish links between immigrant status and disease status [17]. Therefore, this systematic review may partially help to clarify vitamin D-related health deterioration in migrants and moreover, to develop a global guideline that specifies sub-populations, in which the evidence and recommendation might differ from the overall immigrant population.

## Additional files

Additional file 1: Revised PRISMA-P checklist. (DOCX 39 kb)

Additional file 2: Search strategy. (DOCX 34 kb)

## Data Availability

Not applicable

## Abbreviations

25(OH)D: 25-Hydroxyvitamin D serum
A&HCI: Arts and Humanities Citation Index (1975–present)
CIHI: Canadian Institute for Health Information
CPCI-S: Conference Proceedings Citation Index—Science (1990–present)
CPCI-SSH: Conference Proceedings Citation Index—Social Science and Humanities (1990–present)
DRIs: Dietary Reference Intakes
ESCI: Emerging Sources Citation Index (2005–present)
IOM: Institute of Medicine
NICE: National Institute for Health Care Excellence
PECO: Population, Exposure, Comparator, and Outcomes
PRESS: Peer Review of Electronic Search Strategies
PRISMA-P: Preferred Reporting Items for Systematic Reviews and Meta-Analyses Protocol
ROB: Risk of bias tool
ROBINS-E: Risk Of Bias In Non-randomized Studies of Exposure
SCI-EXPANDED: Science Citation Index Expanded (1900–present)
SIGN50: Scottish Intercollegiate Guidelines Network-Publication no. 50
SSCI: Social Sciences Citation Index (1900–present)
WHO: World Health Organization
