# Supplementary material for: Study protocol: Worldwide comparison of vitamin D status of immigrants from different ethnic origins and native-born populations—a systematic review and meta-analysis
Source: Syst Rev. 2019 Aug 22;8:211. doi: 10.1186/s13643-019-1123-4 (PMC6706882; doi:10.1186/s13643-019-1123-4)
Supplement: Supplementary file 2 — Search strategy. (DOCX 34 kb) [file 13643_2019_1123_MOESM2_ESM.docx]

| **#** | **Searches** |
| --- | --- |
| 1 | exp Vitamin D/ |
| 2 | (vitamin D or vitamin D2 or Vitamin D3 or vitamins D or vitamins D2 or Vitamins D3).tw,kf,rn. |
| 3 | ("25(OH)" or 25OH or 25OHD or 25OHD?).tw,kf,rn. |
| 4 | (25OHvitamin? D or 25OHvitamin? D2 or 25OHvitamin? D3).tw,kf,rn. |
| 5 | (cholecalciferol? or colecalciferol? or calciol or vigantol or vigorsan or 1C6V77QF41 or 9VU1KI44GP).tw,kf,rn. |
| 6 | (hydroxycholecalciferol? or hydroxycolecalciferol? or hydroxycalciferol or hydroxyvitamin? or hydroxy vitamin? or hydroxycalcidiol or calcifediol or calcidiol or calderol or d?drogyl or dydrogil or delakmin or hidroferol or rayaldee or u 32070 or u32070 or T0WXW8F54E).tw,kf,rn. |
| 7 | (alfacalcidol or alfarol or alphacalcidol or einsalpha or etalpha or ox?devit? or unalfa or unalpha or URQ2517572).tw,kf,rn. |
| 8 | (dihydroxycholecalciferol? or dihydroxycolecalciferol? or dihydroxyvitamin? or dihydroxy vitamin? or osteo d or ro 21 5816 or ro21 5816 or secalciferol or 0AXX2V8L5Z).tw,kf,rn. |
| 9 | (calcitriol or dihydroxy-20-epi-vitamin? or bocatriol or bonky or calcijex or caraben sc or cicarol or citrihexal or decostriol or dn 101 or dn101 or ecatrol or hitrol or kolkatriol or kosteo or lemytriol or mc 1288 or mc1288 or meditrol or osteotriol or poscal or renatriol or rocaltrol or rexamat or ro 21 5535 or rocaltrol or roical or rolsical or silkis or sitriol or soltriol or tariol or tirocal or triocalcit or vectica or FXC9231JVHl).tw,kf,rn. |
| 10 | (1,25 OHD or 1,25 OHD? or 1,25OHD or 1,25OHD? or 1a,25OHD or 1a,25OHD? or 1a25 OHD or 1a25 OHD?).tw,kf,rn. |
| 11 | (ergocalciferol? or hydroxyergocalciferol or afj d2 or alcovit d2 or aldevit or bentavit or calciferol? or calciferovit or chemovit d or chocola d or condol or d arthrin? or d crivit or d vatine or d vital or d2 vita or davitamon d or davitan or davitin or decaps or dee osterol or dee ron or deeosterol or deeron or dekristol or delta monovit or deltabios or deltalin? or deltamonovit or deltar or deltasterolo or deltavit or deradion or deradione or deratol or dergosten or desyn or desyne or detalup or detamine or deterapion? or devitan or devitil or devitol or di actol or di drol or diactol or dibiovit or didrol or didue vita or diergin? or diferol or difilina or difvitamin d or dilavit or disir or disterin# or divit urto or divitin# or dohyfral d or drisdol or dumovit d or dz idrosol or endo d or ercalciol or ergorone or ergosterid? or ergosterin activatum or ergosterina irradiata or ertron? or feroxyl? or fortedol or fortodyl? or genevis or glicol d2 or idro steral or idrosol d2 or infadin? or infron? or inovitan d or irradiated ergosterol or kalciferol or metadee or min# d2 or mulsiferol or mykostin or mykostine or oldevit or oleovit d2 or oleovitamin d2 or ostelin? or osteodin# or osteovit or osteovitadin? or osteovitin# or ostergil or plivit d or radiamon or radiosterin# or radiostol or radsterin? or raquiferol or ro 850 or shock ferol or shockferol or sinervit d2 or steral or steramin? or sterobiol or sterodin or sterodine or sterogyl or sterosol or sterovit or sterovitin# or ucemine d or ultranol or urto calciosterina or urtosterin# or uvesterol d or vi de or vi di or videlta or vidi or vidiman or vidolen or vidue monico or viduemonico or vigoncal or vio d or viosterin? or viosterol or vitadit or vitaminol or vitaplex or vitasan d or vitastabil? d or vitastabil d or vitasterin? or vitasterol or vitavel d or wandervit d2 or VS041H42XC).tw,kf,rn. |
| 12 | (antitanil or antitetanin? or atecen or calcamin? or calcinosefaktor or dht intensol or dichistrolum or dichyst?rol or dihydral or dihydrotachysterin? or dihydrotachysterol or dihydro tachysterol or dikystrol or dygratyl or hytakerol or manipal or parterol or tachidon or tachystin? or tachystol or tetilan).tw,kf,rn. |
| 13 | exp vitamin d deficiency/ |
| 14 | ((avitaminosis or hypovitaminosis) adj1 (D or D2 or D3)).tw,kf. |
| 15 | (rickets or ricketts or rachiti*).tw,kf. |
| 16 | Osteomalacia?.tw,kf. |
| 17 | ((Osteodystroph* or mineral) adj2 bone disorder?).tw,kf. |
| 18 | or/1-17 |
| 19 | exp "Emigrants and Immigrants"/ |
| 20 | "Emigration and Immigration"/ |
| 21 | "Transients and Migrants"/ |
| 22 | refugees/ |
| 23 | Ethnic Groups/ |
| 24 | minority groups/ |
| 25 | Minority Health/ |
| 26 | ((ethnic* or minority) adj3 (group? or people? or person? population? or patient? or community or communities or individual?)).tw,kf. |
| 27 | (minority adj3 (health or healthcare or ethnic*)).tw,kf. |
| 28 | minorities.tw,kf. |
| 29 | ethnic*.ti. |
| 30 | (alien? or emigrat* or emigrant? or foreigner? or immigrat* or immigrant? or migrant? or migrate? or migration? or migrating or undocumented worker? or foreign born or refugee? or asylum seeker?).tw,kf. |
| 31 | (resettlement? or settlement?).tw,kf. |
| 32 | (displaced adj3 (family or families or individual? or person? or people? or population? or man or men or wom#n or child or children)).tw,kf. |
| 33 | (nationalities or nationality).tw,kf. |
| 34 | or/19-33 |
| 35 | 18 and 34 |
| 36 | animals/ not humans.sh. |
| 37 | 35 not 36 |
| 38 | (comment or editorial or interview or news).pt. |
| 39 | 37 not 38 |
| 40 | 39 use medall |
| 41 | exp vitamin D/ |
| 42 | (vitamin D or vitamin D2 or Vitamin D3 or vitamins D or vitamins D2 or Vitamins D3).tw,kw,rn. |
| 43 | ("25(OH)" or 25OH or 25OHD or 25OHD?).tw,kw. |
| 44 | (25OHvitamin? D or 25OHvitamin? D2 or 25OHvitamin? D3).tw,kw. |
| 45 | (cholecalciferol? or colecalciferol? or calciol or vigantol or vigorsan or 1406-16-2 or 67-97-0).tw,kw,rn. |
| 46 | (hydroxycholecalciferol? or hydroxycolecalciferol? or hydroxycalciferol or hydroxyvitamin? or hydroxy vitamin? or hydroxycalcidiol or calcifediol or calcidiol or calderol or d?drogyl or dydrogil or delakmin or hidroferol or rayaldee or u 32070 or u32070 or 19356-17-3).tw,kw,rn. |
| 47 | (alfacalcidol or alfarol or alphacalcidol or einsalpha or etalpha or ox?devit? or unalfa or unalpha or 41294-56-8).tw,kw,rn. |
| 48 | (dihydroxycholecalciferol? or dihydroxycolecalciferol? or dihydroxyvitamin? or dihydroxy vitamin? or osteo d or ro 21 5816 or ro21 5816 or secalciferol or 40013-87-4 or 55721-11-4).tw,kw,rn. |
| 49 | (calcitriol or dihydroxy-20-epi-vitamin? or bocatriol or bonky or calcijex or caraben sc or cicarol or citrihexal or decostriol or dn 101 or dn101 or ecatrol or hitrol or kolkatriol or kosteo or lemytriol or mc 1288 or mc1288 or meditrol or osteotriol or poscal or renatriol or rocaltrol or rexamat or ro 21 5535 or rocaltrol or roical or rolsical or silkis or sitriol or soltriol or tariol or tirocal or triocalcit or vectica or 32222-06-3 or 32511-63-0 or 66772-14-3).tw,kw,rn. |
| 50 | (1,25 OHD or 1,25 OHD? or 1,25OHD or 1,25OHD? or 1a,25OHD or 1a,25OHD? or 1a25 OHD or 1a25 OHD?).tw,kw,rn. |
| 51 | (ergocalciferol? or hydroxyergocalciferol or afj d2 or alcovit d2 or aldevit or bentavit or calciferol? or calciferovit or chemovit d or chocola d or condol or d arthrin? or d crivit or d vatine or d vital or d2 vita or davitamon d or davitan or davitin or decaps or dee osterol or dee ron or deeosterol or deeron or dekristol or delta monovit or deltabios or deltalin? or deltamonovit or deltar or deltasterolo or deltavit or deradion or deradione or deratol or dergosten or desyn or desyne or detalup or detamine or deterapion? or devitan or devitil or devitol or di actol or di drol or diactol or dibiovit or didrol or didue vita or diergin? or diferol or difilina or difvitamin d or dilavit or disir or disterin# or divit urto or divitin# or dohyfral d or drisdol or dumovit d or dz idrosol or endo d or ercalciol or ergorone or ergosterid? or ergosterin activatum or ergosterina irradiata or ertron? or feroxyl? or fortedol or fortodyl? or genevis or glicol d2 or idro steral or idrosol d2 or infadin? or infron? or inovitan d or irradiated ergosterol or kalciferol or metadee or min# d2 or mulsiferol or mykostin or mykostine or oldevit or oleovit d2 or oleovitamin d2 or ostelin? or osteodin# or osteovit or osteovitadin? or osteovitin# or ostergil or plivit d or radiamon or radiosterin# or radiostol or radsterin? or raquiferol or ro 850 or shock ferol or shockferol or sinervit d2 or steral or steramin? or sterobiol or sterodin or sterodine or sterogyl or sterosol or sterovit or sterovitin# or ucemine d or ultranol or urto calciosterina or urtosterin# or uvesterol d or vi de or vi di or videlta or vidi or vidiman or vidolen or vidue monico or viduemonico or vigoncal or vio d or viosterin? or viosterol or vitadit or vitaminol or vitaplex or vitasan d or vitastabil? d or vitastabil d or vitasterin? or vitasterol or vitavel d or wandervit d2 or 50-14-6 or 50809-47-7 or 8042-78-2).tw,kw,rn. |
| 52 | (antitanil or antitetanin? or at 10 or at10 or atecen or calcamin? or calcinosefaktor or dht intensol or dichistrolum or dichyst?rol or dihydral or dihydrotachysterin? or dihydrotachysterol or dihydro tachysterol or dikystrol or dygratyl or hytakerol or manipal or parterol or tachidon or tachystin? or tachystol or tetilan or 67-96-9).tw,kw,rn. |
| 53 | vitamin D deficiency/ |
| 54 | exp rickets/ |
| 55 | osteomalacia/ |
| 56 | ((avitaminosis or hypovitaminosis) adj1 (D or D2 or D3)).tw,kw. |
| 57 | (rickets or ricketts or rachiti*).tw,kw. |
| 58 | Osteomalacia?.tw,kw. |
| 59 | ((Osteodystroph* or mineral) adj2 bone disorder?).tw,kw. |
| 60 | or/41-59 |
| 61 | exp migrant/ |
| 62 | exp migration/ |
| 63 | ethnic group/ |
| 64 | ancestry group/ |
| 65 | minority group/ |
| 66 | minority health/ |
| 67 | ((ethnic* or minority) adj3 (group? or people? or person? population? or patient? or community or communities or individual?)).tw,kw. |
| 68 | (minority adj3 (health or healthcare or ethnic*)).tw,kw. |
| 69 | minorities.tw,kw. |
| 70 | ethnic*.ti. |
| 71 | (alien? or emigrat* or emigrant? or foreigner? or immigrat* or immigrant? or migrant? or migrate? or migration? or migrating or undocumented worker? or foreign born or refugee? or asylum seeker?).tw,kw. |
| 72 | (resettlement? or settlement?).tw,kw. |
| 73 | (displaced adj3 (family or families or individual? or person? or people? or population? or man or men or wom#n or child or children)).tw,kw. |
| 74 | (nationalities or nationality).tw,kw. |
| 75 | or/61-74 |
| 76 | 60 and 75 |
| 77 | exp animal/ or exp animal experimentation/ or exp animal model/ or exp animal experiment/ or nonhuman/ or exp vertebrate/ |
| 78 | exp human/ or exp human experimentation/ or exp human experiment/ |
| 79 | 77 not 78 |
| 80 | 76 not 79 |
| 81 | editorial.pt. |
| 82 | 80 not 81 |
| 83 | 82 use emczd |
| 84 | 40 or 83 |
| 85 | remove duplicates from 84 |
